# Mimicking Cardiac Fibrosis in a Dish: Fibroblast Density Rather than Collagen Density Weakens Cardiomyocyte Function

**DOI:** 10.1007/s12265-017-9737-1

**Published:** 2017-03-09

**Authors:** Ariane C.C. van Spreeuwel, Noortje A.M. Bax, Bastiaan J. van Nierop, Annemieke Aartsma-Rus, Marie-José T.H. Goumans, Carlijn V.C. Bouten

**Affiliations:** 1 Department of Biomedical Engineering, Eindhoven University of Technology, PO Box 513, Eindhoven, 5600 MB the Netherlands; 2 Institute for Complex Molecular Systems, Eindhoven University of Technology, Eindhoven, the Netherlands; 3 Department of Human Genetics, Leiden University Medical Center, Leiden, the Netherlands; 4 Department of Molecular Cell Biology, Leiden University Medical Center, Leiden, the Netherlands

**Keywords:** Cardiac fibrosis, Engineered cardiac tissue, Cardiac fibroblast proliferation, ECM accumulation, Cardiomyocyte functionality

## Abstract

Cardiac fibrosis is one of the most devastating effects of cardiac disease. Current in vitro models of cardiac fibrosis do not sufficiently mimic the complex in vivo environment of the cardiomyocyte. We determined the local composition and mechanical properties of the myocardium in established mouse models of genetic and acquired fibrosis and tested the effect of myocardial composition on cardiomyocyte contractility in vitro by systematically manipulating the number of fibroblasts and collagen concentration in a platform of engineered cardiac microtissues. The in vitro results showed that while increasing collagen content had little effect on microtissue contraction, increasing fibroblast density caused a significant reduction in contraction force. In addition, the beating frequency dropped significantly in tissues consisting of 50% cardiac fibroblasts or higher. Despite apparent dissimilarities between native and in vitro fibrosis, the latter allows for the independent analysis of local determinants of fibrosis, which is not possible in vivo*.*

**Electronic supplementary material:**

The online version of this article (doi:10.1007/s12265-017-9737-1) contains supplementary material, which is available to authorized users.

## Introduction

The heart consists of cardiomyocytes, fibroblasts, and other cardiac cells that are supported by a complex meshwork of fibers, the cardiac extracellular matrix (ECM). In case of cardiac disease, the heart is subject to changes in composition and structure, often leading to cardiac fibrosis. While at first, fibrogenesis is an effective mechanism of tissue repair, ongoing adverse remodeling will lead to a decrease in cardiac functionality and eventually to the development of heart failure [1]. To halt or reverse this process, anti-fibrotic therapies are being developed [2], so far with limited success, due to the fact that the extent and distribution of fibrosis vary according to the underlying pathology. Furthermore, there is a lack of knowledge about the effects of fibrosis at the cellular level [3]. Engineered cardiac tissues are excellent models to mimic and study normal and diseased cardiac development and physiology and therefore open new avenues for therapy assessment. The tissues can be cultured under highly controlled conditions and give insights into the responses of cells and ECM on isolated biochemical and biophysical stimuli, which would be impossible to study in vivo. To apply such tissue models as therapy screening platforms, it is important that the models accurately resemble the in vivo characteristics of the disease and that in vitro results correlate with in vivo outcomes [4].

To our knowledge, no engineered tissue model of cardiac fibrosis has been reported that takes into account this in vivo to in vitro translation. Therefore, we aimed to develop an engineered microtissue platform that mimics various aspects of cardiac fibrosis based on the analysis of two well-established mouse models of cardiac fibrosis having either a genetic or an acquired cause of cardiac disease. By comparing the in vitro outcomes to the native situation, strengths and weaknesses of such disease models could be identified.

Current engineered cardiac disease models mainly incorporate genetically affected cells to capture a cardiac disorder [5, 6]. These models primarily focus on intrinsic problems of the cardiomyocytes, while for cardiac fibrosis, the ECM and the cardiac fibroblasts are of critical importance. To date, only few in vitro models of cardiac fibrosis have been developed [7–9]. Unfortunately, most of these models are 2D co-cultures and thus lack the important 3D environment of a fibrotic ECM [7, 8]. For cardiac cells, it has been shown that 3D cultures more closely mimic natural tissue environments compared to 2D cultures, resulting in different outcomes of proliferation, attachment to the ECM and maturation in 2D compared to 3D cultures [10]. Galie et al. [9] cultured cardiac fibroblasts in a 3D collagen gel to investigate the paracrine effect of mesenchymal stem cell injection in a scar tissue. Although ECM was incorporated in this model of fibrosis, cardiomyocytes were lacking. Our engineered microtissues include cardiomyocytes, cardiac fibroblasts, and ECM, since all are affected by fibrosis.

Cardiac fibrosis is generally referred to as an increase in the number of fibroblasts in the heart which lead to the excessive production and deposition of several extracellular matrix proteins, mainly collagen type I. Yet, different forms of fibrosis can be recognized, which all have their own characteristics.

Reactive fibrosis, for instance, has mostly been described in patients with hypertension and diabetes mellitus, but is also present in the aging heart and in hearts suffering from pressure overload. An important characteristic of this type of fibrosis is the interstitial increase in ECM content and absence of cell loss [11, 12]. Replacement or scarring fibrosis on the contrary corresponds to the local replacement of cardiomyocytes by fibrosis after cell death, for example after myocardial infarction [11, 12].

Due to this diversity, cardiac fibrosis is such a complex condition that the relative importance of all different aspects that play a role cannot be studied at once. Therefore, our microtissue platform allows for the independent analysis of different aspects of fibrosis, hence mimicking a variety of cardiac fibrotic pathologies.

Mechanical properties, as well as composition of the ECM of the two mouse models for cardiac pathologies accompanied with fibrosis, were analyzed to obtain detailed in vivo aspects. Although multiple mouse models for cardiac disease are available in this study, the mdx and TAC model were chosen because the origin and development of fibrosis in these two models is different. Secondly, both mouse models are frequently described in literature and often used to test anti-fibrotic therapies [12–16]. The *mdx* mouse was chosen as a model of genetic cardiac disease [17, 18], while a mouse model with transverse aortic constriction (TAC) was chosen to represent acquired heart disease [19]. Both mouse models are known to develop cardiac fibrosis, based on general histological methods for collagen assessment [15, 17]. Mechanical properties as well as localization, spatial distribution, and composition of the fibrotic areas in the heart were determined and used as input for the in vitro tissue model. Secondly, since activation and proliferation of fibroblasts is a well-known contributor of cardiac fibrosis, especially after TAC [20, 21], increase in fibroblast number was also incorporated as aspect of fibrosis. To this end, mouse neonatal cardiac cells were cultured in engineered cardiac microtissues [22]. To mimic various aspects of fibrosis, either the collagen content or the number of cardiac fibroblasts was systematically increased. Mechanical properties and matrix composition of the in vitro microtissues were quantified and compared to the obtained in vivo dataset. Contraction force and beating frequency of the cardiac microtissues were determined to study the correlation between fibrosis and cardiac contractile function.

In this tunable microtissue platform based on in vivo aspects of fibrosis, both the increase of collagen content and fibroblast number that occur with development of cardiac fibrosis were mimicked independently. The in vitro results showed that while increasing collagen content had little effect on microtissue contraction, increasing fibroblast density caused a significant reduction in contraction force. In addition, the beating frequency dropped significantly in tissues consisting of 50% cardiac fibroblasts or higher. Hereby, we were able to show that increased fibroblast density has a more detrimental effect on cardiomyocyte contractile function than accumulation of collagen. Furthermore, this model system can be easily adapted to mimic different stages and forms of cardiac fibrosis and thereby opens new opportunities for development of effective anti-fibrotic treatments.

## Methods

### Preparation and Analysis of Cardiac Tissue from TAC and *mdx* Mouse Models

C57Bl/10ScSnJ (control) and *mdx* (C57Bl/10ScSn-DMD^mdx^/J) male mice at 10 months were sacrificed by cervical dislocation and hearts were isolated [17, 18]. TAC hearts were excised 9 weeks after surgery as well as the hearts from age matched male controls (C57BL/6). Cardiac tissue was then processed for further analysis of mechanical properties and tissue composition as outlined in the supplementary material.

### In Vitro Microtissue Seeding and Analysis

Mouse neonatal cardiomyocytes and cardiac fibroblasts were isolated from 1- to 3–day-old C57/BL6 mouse hearts as described before [22, 23], and most cardiac fibroblasts were removed to obtain an enriched cardiomyocyte population. Further details are provided in the supplementary material. Microfabricated tissue gauges (μTUG) with microposts as uniaxial tissue constraints were fabricated using soft lithography [22, 24]. To create microtissues with increasing fibroblast density, hydrogel composition was the same as described previously [22], resulting in a final collagen concentration of 1.6 mg/ml. For microtissues with increasing collagen concentration, the final concentration was varied between 0.5, 1.5, 2.5, and 3.5 mg/ml collagen type I. For both groups, cells were trypsinized and suspended in the hydrogel at a concentration of 1 × 10^6^ cells/ml. For microtissues with increasing collagen content, only enriched cardiomyocytes were used, while for microtissues with extra cardiac fibroblasts, this cell suspension consisted of enriched cardiomyocytes mixed with 0, 15, 30, and 45% cardiac fibroblasts. At different time points, microtissues were processed for further analysis of mechanical properties, composition, and contractility as described in the supplementary material.

## Results

### Composition and Mechanical Properties of Patchy Fibrosis in *mdx* and TAC Mouse Hearts

**Fig. 1:**
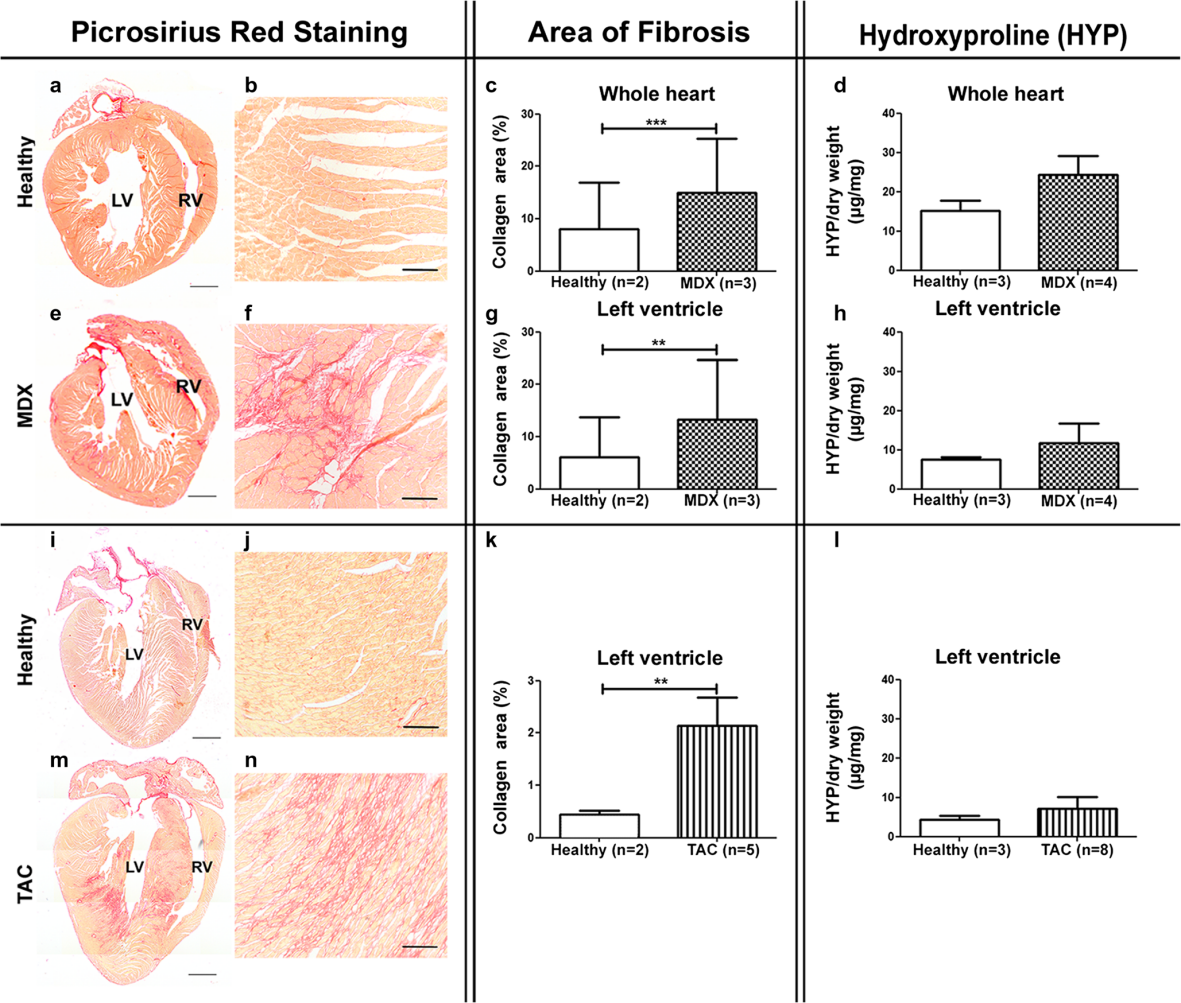
Myocardial fibrosis in *mdx* and TAC mouse hearts. Low-power photomicrographs of picrosirius red-stained sections of healthy (*a*) and *mdx* (*e*) transgenic hearts from mice aged 10 months and healthy (*i*) and TAC hearts 9 weeks after constriction (*m*) show patchy fibrosis in both disease models. Enlargement pictures of healthy (*b*, *j*) and fibrotic myocardium (*f*, *n*). Bar graphs represent areas of collagen (%) determined from the picrosirius red staining of the whole heart (*c*) and left ventricle (*g*, *k*) and show a significant increase of collagen for both disease models. Hydroxyproline content is not significantly different between healthy and diseased whole heart (*d*) or left ventricle (*h*, *l*). *Scale bar a*/*e*/*i*/*m* = 1000 μm and *b*/*f*/*j*/*n* = 100 μm. **P < 0.01 and ***P < 0.001

To obtain an overview of the degree of fibrosis that was developed in the *mdx* and TAC hearts, picrosirius red staining was performed. Histological analysis showed that collagen was present throughout the whole heart of *mdx* mice (Fig. 1a–f, i–n). Most severe fibrosis was observed in the entire right ventricle. Interestingly, in the left ventricle, only patches of interstitial fibrosis were observed. Furthermore, semi-quantitative image analysis revealed a significantly larger area of collagen present in *mdx* hearts compared to healthy hearts (1.8× higher) (Fig. 1c). For the left ventricle alone, the collagen area of *mdx* left ventricles was 1.7× larger than in healthy left ventricles (Fig. 1g).

Histological analysis of hearts from mice 9 weeks after TAC showed perivascular fibrosis and patchy interstitial fibrosis. Patchy fibrosis was especially found in the left ventricle and ventricular septum, while the right ventricles seemed normal. 4.8× more collagen was found in TAC left ventricles compared to healthy left ventricles (Fig. 1k).

To check whether this local increase of collagen detected with histology also affected total collagen content, a HYP assay was performed. Whole hearts of *mdx* mice contained 1.6× more HYP per milligram dry weight of tissue when compared to hearts of healthy mice, although this was not significantly different (Fig. 1d). Furthermore, no significant differences in the amount of HYP were found in the left ventricles of *mdx* mice when compared to healthy ventricles (Fig. 1h). Similarly, for the left ventricles of TAC hearts, a non-significant increase of HYP was found, with 1.6× more HYP per milligram dry weight of tissue in TAC left ventricles when compared to control left ventricles (Fig. 1l).

**Fig. 2:**
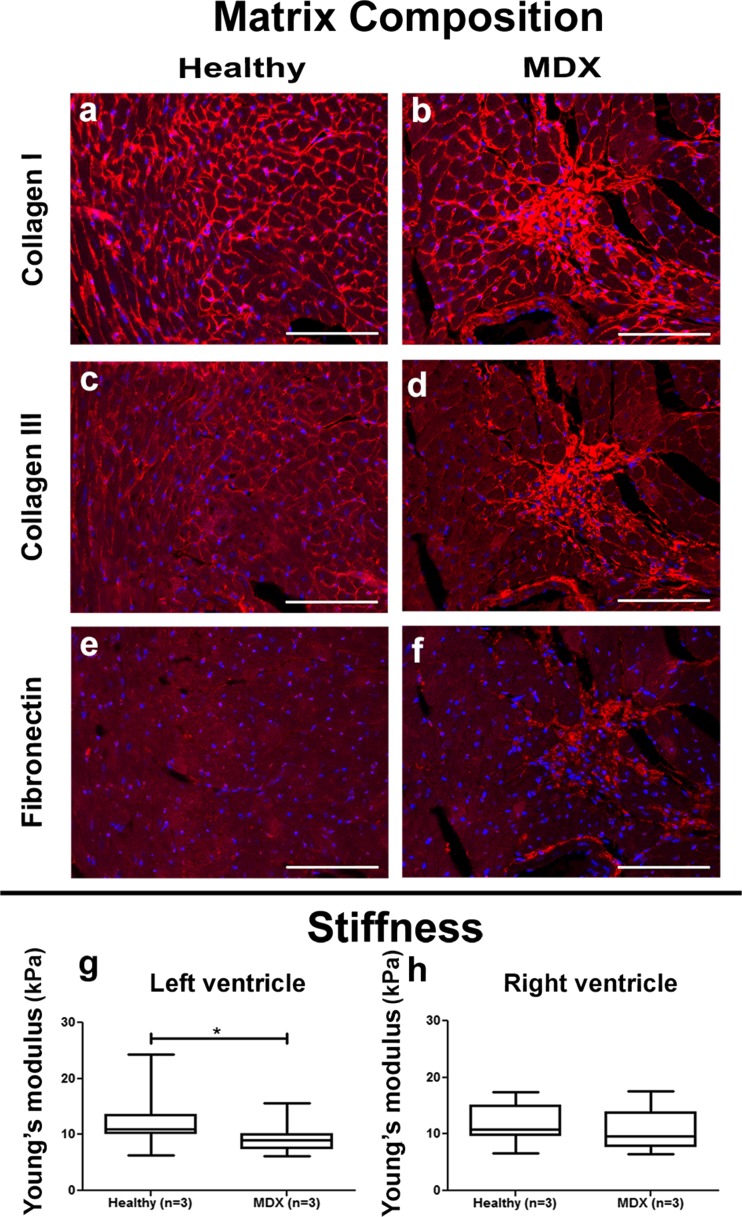
Matrix composition and stiffness of healthy and *mdx* hearts. Representative pictures of myocardial tissue of healthy and *mdx* left ventricles show accumulation of collagen I (*a*, *b*) collagen III (*c*, *d*) and fibronectin (*e*, *f*) in the fibrotic areas. Nuclei are stained in *blue* with DAPI. Left ventricular stiffness of *mdx* hearts is significantly lower than healthy hearts (*g*), while stiffness is not significantly different between healthy and *mdx* right ventricles (*h*). *Scale bar* = 50 μm. *P < 0.05

**Fig. 3:**
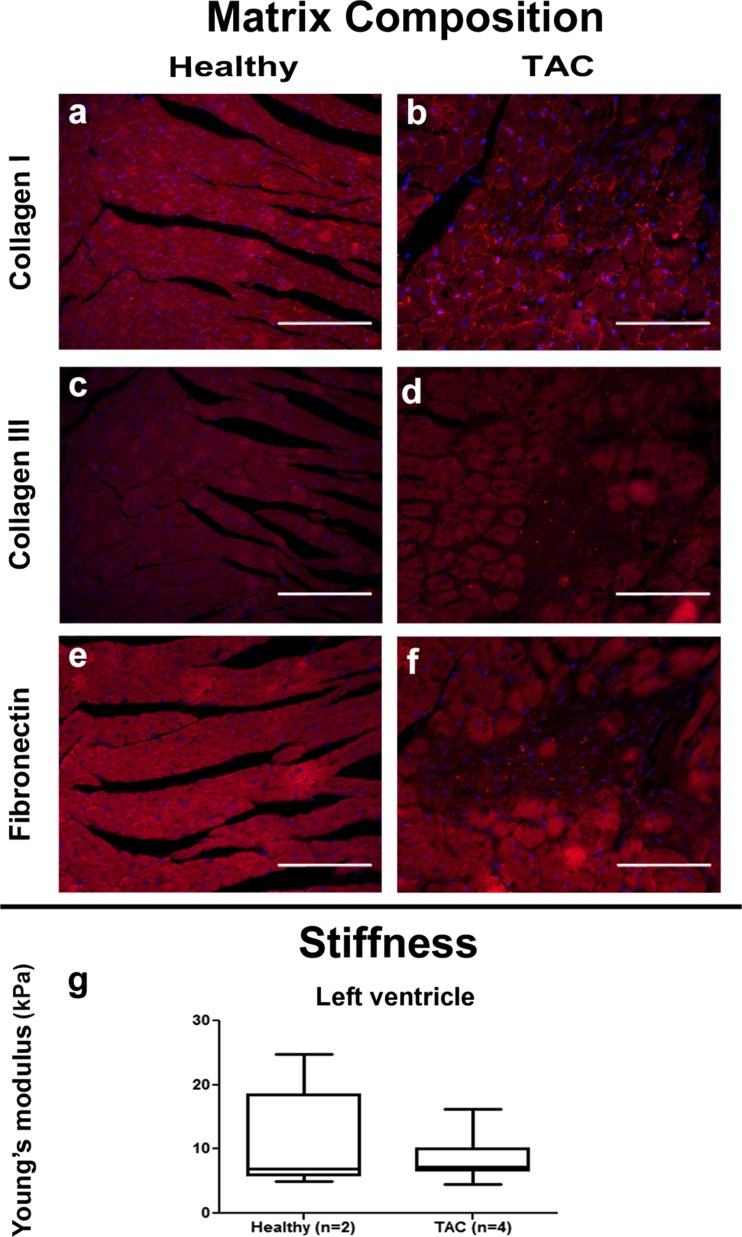
Matrix composition and stiffness of healthy and TAC hearts. Presence of collagen I (*a*, *b*) but not of collagen III (*c*, *d*) and fibronectin (*e*, *f*) is shown in representative pictures of fibrotic areas of in the myocardial tissue. Nuclei are stained in *blue* with DAPI. Left ventricular stiffness is not significantly different between healthy and TAC hearts (*g*). *Scale bar* = 50 μm

Immunohistological analysis for collagen I and III and fibronectin was performed on the fibrotic areas of both *mdx* and TAC left ventricles. Fibrotic patches in *mdx* left ventricles showed presence of collagen I and III and fibronectin (Fig. 2a–f). In contrast, the fibrotic areas of TAC left ventricles showed only presence of collagen I and not of collagen III or fibronectin (Fig. 3a–f).

To determine if the formation of these patchy areas of fibrosis affected the stiffness of the myocardium, indentation tests were performed on tissue samples of the left ventricle. Young’s modulus of *mdx* left ventricles (9.4 ± 2.6 kPa) was significantly lower than healthy left ventricles (12.4 ± 4.5 kPa) (Fig. 2g), while the stiffness of the right ventricle was not significantly different between *mdx* (10.8 ± 3.6 kPa) and control hearts (11.9 ± 3.1 kPa) (Fig. 2h). For the TAC hearts, Young’s modulus of the left ventricles (8.2 ± 2.7 kPa) was not significantly different when compared to healthy left ventricles (11.1 ± 7.4 kPa) (Fig. 3g).

### In Vitro Tissue Model of Cardiac Fibrosis

**Fig. 4:**
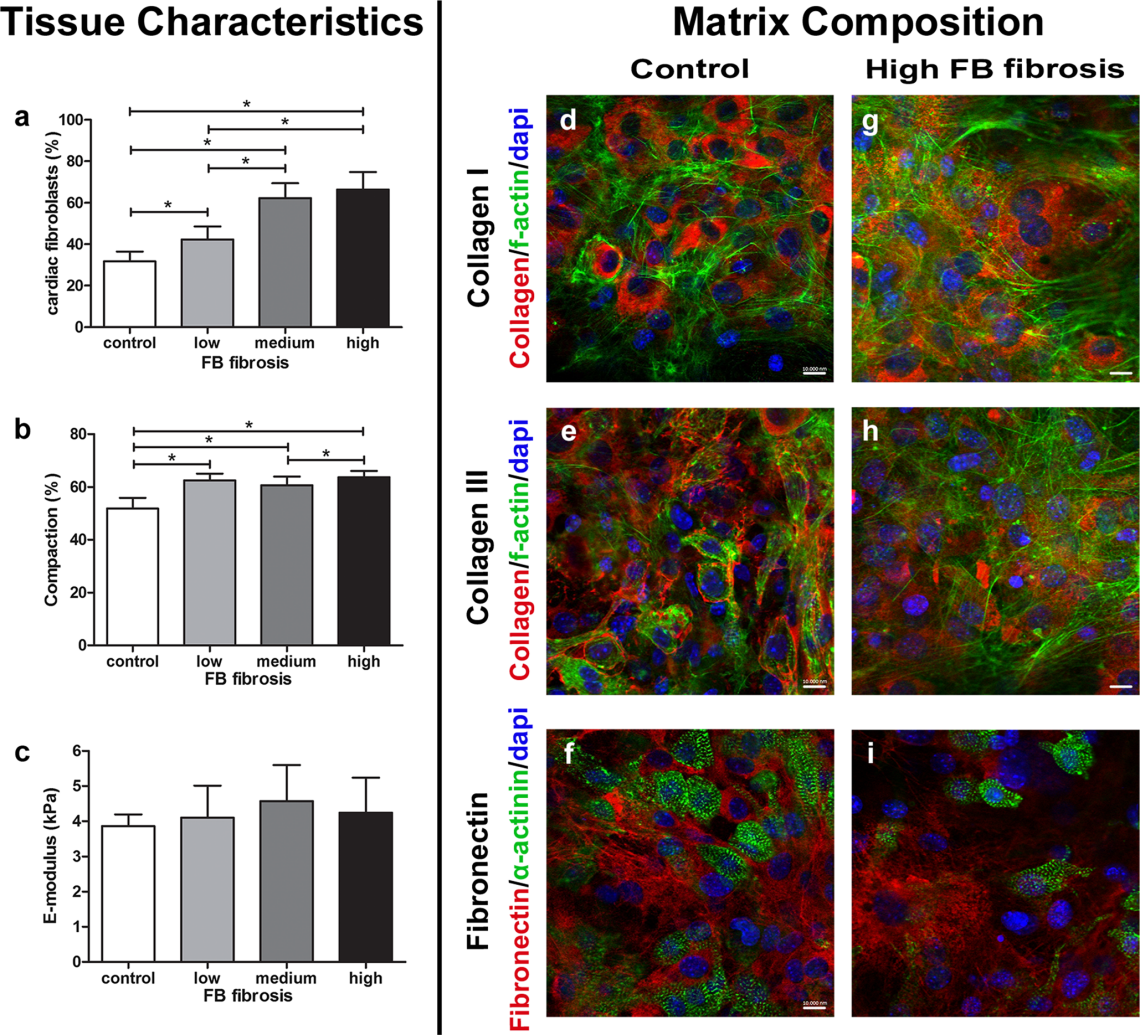
Cellular and matrix composition, compaction and stiffness of FB fibrosis microtissues. Increasing fibroblast percentage (*a*) was confirmed using immunohistochemistry markers for cardiomyocytes (α-actinin) and cardiac fibroblasts (vimentin). Compaction of the microtissues increased with increasing FB fibrosis (*b*). Stiffness remained constant for increasing fibroblast number (*c*). Representative pictures of control and FB fibrosis microtissues show homogeneous distribution of collagen I (*d*, *g*), collagen III (*e*, *h*) and fibronectin (*f*, *i*) in all microtissues. Fibronectin is predominantly present in the cytoplasm of sarcomeric α-actinin (*green*) negative cells (*f*, *i*). *Error bars* for fibroblast percentage and compaction represent SD of *N* ≥ 30 from three independent experiments. *Error bars* for E-modulus represent nine measurement spots on three samples in one experiment. *Scale bar* = 10 μm (*d*–*i*). *P < 0.05

The different features of cardiac fibrosis were mimicked by manipulating the number of fibroblasts (referred to as FB fibrosis) or the collagen concentration (referred to as ECM fibrosis) in cardiac microtissues seeded in a previously develop μTUG system [22]. Enriched cardiomyocytes were used to create cardiac microtissues for the FB control group, resulting in tissues with 68 ± 5% cardiomyocytes and 32 ± 5% cardiac fibroblasts (Fig. 4a). These numbers were achieved by counting sarcomeric α-actinin positive cardiomyocytes and vimentin-positive cardiac fibroblasts. To increase fibroblast density, cardiac fibroblasts from a previous isolation were added to the enriched cardiomyocytes, resulting in microtissues with a range of fibroblast densities. Next to the FB control group with 32 ± 5% fibroblasts, adding extra fibroblasts resulted in microtissues with 42 ± 6, 62 ± 7, and 66 ± 8% fibroblasts, which are further referred to as the low, medium, and high FB fibrosis group respectively (Fig. 4a). Increasing the number of fibroblasts affected the compaction of the microtissues. The control group compacted by 52 ± 4%, while adding extra fibroblasts significantly increased the compaction up to 64 ± 2% for the high FB fibrosis group (Fig. 4b). After 7 days of culture, cardiac microtissues of the FB control group showed presence of collagen I throughout the microtissues and also around the nuclei of both cardiomyocytes and fibroblast (Fig. 4d). Although no exogenous collagen III and fibronectin were added, both matrix components were homogeneously distributed throughout the microtissues and were also present in the cytoplasm of both cell types (Fig. 4e, f). Fibronectin was predominantly present in the cytoplasm of sarcomeric α-actinin-negative fibroblasts (Fig. 4f). To verify if the increase in cardiac fibroblasts also affected matrix composition, microtissues of the high FB fibrosis group were also analyzed for presence of collagen I and III and fibronectin (Fig. 4g–i). The high FB fibrosis group contained much more fibroblasts than the control group, as indicated by the decrease in sarcomeric α-actinin-positive cells (Fig. 4i). However, the increase in cardiac fibroblasts did not result in changes in the presence of collagen I and III and fibronectin between the control and high FB fibrosis groups (Fig. 4d–i). Stiffness of the different tissue compositions of all four FB fibrosis groups was determined by indentation. Young’s modulus was 3.9 ± 0.3 kPa for the FB control group and did not show significant changes for tissues with increasing fibroblast content (Fig. 4c).

**Fig. 5:**
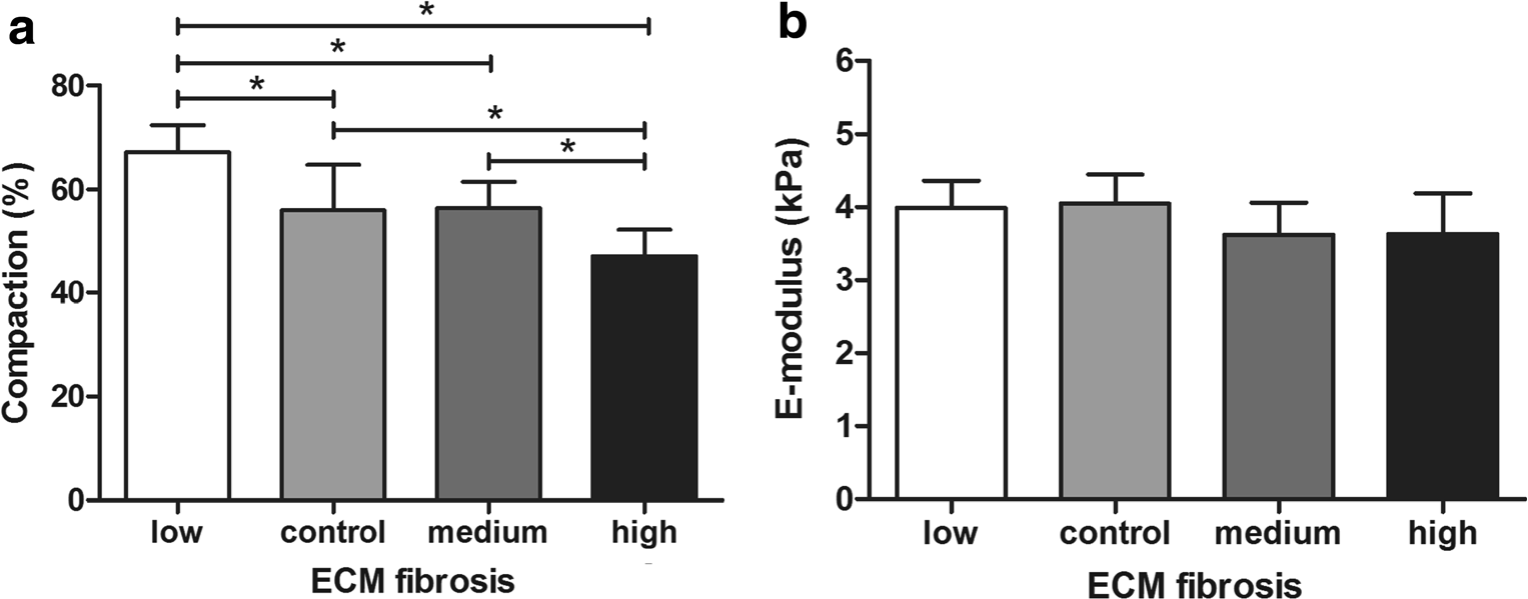
Compaction and stiffness of ECM fibrosis microtissues. Compaction of the microtissues decreased with increasing collagen concentration (*a*), while stiffness remained constant (*b*). *Error bars* for compaction represent SD of *N* ≥ 40 from three independent experiments. *Error bars* for E-modulus represent nine measurement spots on three samples in one experiment. *P < 0.05

Next to manipulating the fibroblast content of the cardiac microtissues, we also varied the collagen concentration of the microtissues to mimic fibrosis. Increased collagen concentration was accompanied by a reduction in compaction of the tissues. The low ECM fibrosis group compacted for 67 ± 5% and increasing collagen concentration reduced the compaction to 47 ± 5% for the high ECM fibrosis group (Fig. 5a). Stiffness of the ECM control group was 4.0 ± 0.4 kPa and did not significantly change for tissues with increasing collagen concentration (Fig. 5b).

### Contractility of Fibrotic Cardiac Microtissues

**Fig. 6:**
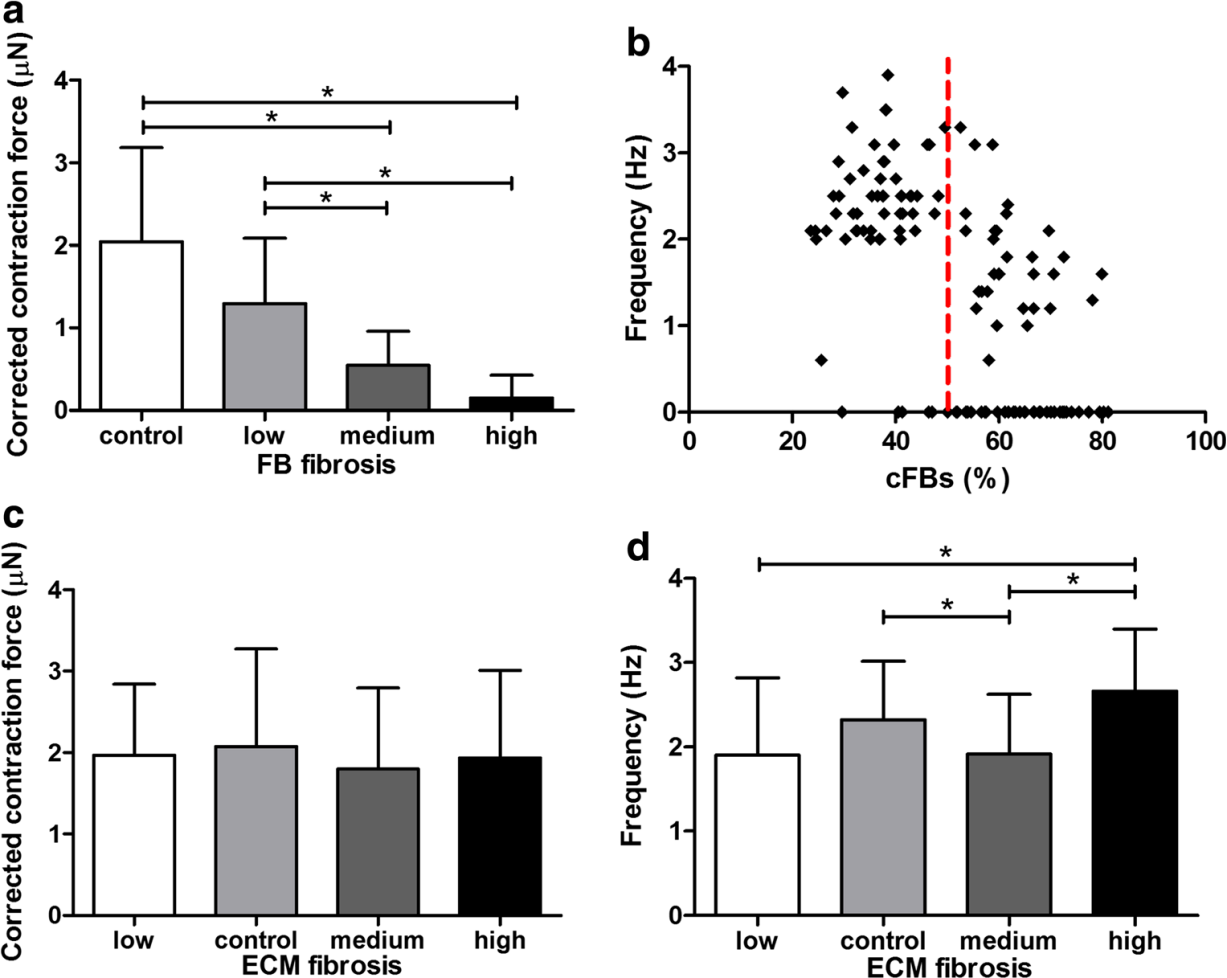
Effect of FB and ECM fibrosis on microtissues contractility. Contraction force corrected for compaction (*a*) decreased with increasing FB fibrosis. A threshold of 50% cardiac fibroblasts was found (indicated by the *red line*) after which beating of the FB fibrosis microtissues was severely hampered (*b*). On the contrary, ECM fibrosis had little effect on microtissues contractility. Corrected contraction force was similar for all ECM fibrosis groups (*c*). Beating frequency fluctuated but showed no increasing or decreasing trend (*d*). *Error bars* represent SD of *N* ≥ 30 from three independent experiments. *P < 0.05

Beating frequency and dynamic contraction force of the microtissues were measured to study the effect of both FB and ECM fibrosis on cardiomyocyte contractility separately. Dynamic contraction force was determined at 2.0 ± 1.1 μN for the FB control group and reduced to 1.3 ± 0.8 μN for the low FB fibrosis group, although this decrease was not significant (Fig. 6a). Interestingly, further increase in fibroblast density did cause a significant decrease in contraction force to 0.5 ± 0.4 μN (medium FB fibrosis) and 0.1 ± 0.3 μN (high FB fibrosis), resulting in almost no contraction of microtissues in the high FB fibrosis group (Fig. 6a). Next to contraction force, beating frequency is also an important indicator of contractility. Beating frequency of the FB control group was 2.2 ± 0.7 Hz. For the low FB fibrosis group, beating frequency was not significantly affected. However, beating frequency of the medium and high FB fibrosis group significantly reduced to 1.0 ± 0.9 and 0.3 ± 0.5 Hz. The fact that the beating frequency only decreased for the medium and high FB fibrosis group raised the suggestion of a threshold in fibroblast density above which beating of the microtissues is drastically reduced. Indeed, analysis of frequency and fibroblast percentage of each individual microtissues showed a clear threshold at 50% cardiac fibroblasts, above which the average beating frequency dropped significantly. Microtissues with less than 50% fibroblasts had an average frequency of 2.3 ± 0.9 Hz with only 8% non-beating tissues compared to 0.6 ± 0.9 Hz with 62% non-beating tissues for microtissues with more than 50% cardiac fibroblasts (Fig. 6b).

By increasing the collagen concentration of the microtissues, another aspect of cardiac fibrosis was mimicked. Surprisingly, increasing collagen content had no effect on contraction force or beating frequency (Fig. 6c, d). Contraction force was similar for all four groups (Fig. 6c). Beating frequency of the microtissues fluctuated between 1.9 ± 0.9 and 2.7 ± 0.7 Hz with significant differences but no increasing or decreasing trend (Fig. 6d). This implies that the cardiomyocytes were still able to exert the same force of contraction, even though there was more collagen in between neighboring cells.

## Discussion

### Cardiac Fibrosis in *mdx* and TAC Mouse Hearts

Histological analysis confirmed the presence of fibrosis in hearts from mice with both genetic and acquired heart disease. In *mdx* mice, fibrosis in the left ventricle was characterized by a patchy distribution whereas fibrosis was homogeneously distributed throughout the entire right ventricle. In earlier studies, patchy fibrosis was shown to affect all regions of the left and right ventricle approximately equal [25], while others described fibrosis in *mdx* hearts for the whole heart or left ventricle only, thereby omitting conclusions about the distribution pattern [17, 18, 26]. Current detailed analysis of the fibrotic lesions showed presence of collagen I and III as well as increased expression of fibronectin. While increase in fibronectin in fibrotic areas is well documented in skeletal muscle and diaphragms of *mdx* mice [27], to our knowledge, this is the first time that the presence of fibronectin in fibrotic lesions in *mdx* hearts is described. In TAC hearts, perivascular fibrosis and interstitial patchy fibrosis was observed throughout the left ventricle. Patchy fibrosis was previously described from 16 weeks onwards after TAC, while at earlier time points, only diffuse fibrosis was reported [28, 29].

Although immunohistochemistry revealed presence of fibrosis for both *mdx* and TAC hearts, biochemical analysis of HYP did not show any increase in collagen content. The contradiction in this data might be related to the sensitivity of both techniques. HYP is a major component of collagen, but only comprises 13.5% of the amino acid composition [30–32]. This leads to lower total collagen content verified when using biochemical assays since the affinity is less. Picrosirius red on the other hand binds with high affinity to the triple helix structure found in all collagen fibers leading to an over-estimate of the total amount of collagen [32], especially when patchy fibrosis is present. Another explanation might be related to the fact that this biochemical assay is a global measure while there is only an increase in collagen in the fibrotic patches and not in the rest of the hearts.

It is generally assumed that accumulation of ECM components increases tissue stiffness. Surprisingly, indentation tests showed no differences in ventricular stiffness for TAC hearts and a significant decrease in left ventricular stiffness of *mdx* hearts, even though patches of fibrosis with an increase in matrix proteins are detected in the left ventricle. However, for skeletal muscle lower Young’s moduli have been reported for *mdx* mice when compared to healthy mice [33, 34]. This decrease in stiffness could be attributed to loss of actin filaments and/or microtubules [30], or cardiomyocyte death [35]. Collapse of the ECM due to cardiomyocyte death may result in patchy fibrosis even though there is no real increase in matrix proteins and thereby no increase in myocardial stiffness.

### Cardiac Fibrosis Mimicked in Engineered Microtissues

A homogeneous distribution of collagen I, collagen III, and fibronectin was found in control microtissues and after 7 days of culture ECM components were also expressed in the cytoplasm of the cardiac cells. Furthermore, microtissues from the high FB fibrosis group showed the same distribution of these proteins and no increase or decrease could be observed. Previous work by Kelm et al. characterized the matrix of myocardial microtissues from primary neonatal mouse cardiomyocytes in hanging drop cultivations and showed presence of fibronectin but absence of collagen I after 7 days in culture [36]. The absence of collagen I in these purified mouse cardiomyocyte cultures was explained as the predominant production of collagen I by fibroblasts [36].

Changing tissue properties such as the cardiomyocyte/fibroblast ratio or the collagen content could also affect the mechanical properties of the cardiac microtissues. For native, cardiac tissue is has been shown that the stiffness varies between healthy and infarct areas of the myocardium [37]. Some studies assumed that tissue stiffness increases when collagen content increases [38], which is contradicted by our results that show similar Young’s moduli for all groups. However, recently it has been shown that not collagen content but collagen cross-linking determines tissue stiffness [39, 40]. Due to the short culture time, we assume that cross-linking in our microtissues is still limited and therefore will not affect tissue stiffness. This is in line with previous research by Smith et al. who showed that increased collagen content without increased cross-linking density did not affect tissue stiffness [39]. Another explanation for this surprising result might be related to the fact that indentation in this study was essential to perform non-destructive mechanical characterization of the living cell containing microtissue while being attached to the microposts. The cells compacted the hydrogels thereby contributing to overall cell-gel construct density and stiffness, especially in the uniaxial constrained configuration on our micropost system. This contribution may have overruled the change in collagen concentration.

Although in vitro tissue models are always a simplified version of the native complexity, it is important to include the most important building blocks of the tissue and the specific characteristics of the disease of interest. Therefore, our microtissue model consists of an ECM containing hydrogel with a mixture of cardiomyocytes and cardiac fibroblasts. In our in vitro model of cardiac fibrosis, only an increase of collagen type I was mimicked as observed in the fibrotic patches of TAC hearts. *Mdx* hearts also showed patchy fibrosis in the left ventricle with increased collagen I, but also collagen III and fibronectin were increased. Off course, exogenous collagen III and fibronectin could be added to the hydrogel composition to increase the resemblance to the native situation. There is still a mismatch between the mechanical properties of the in vitro model and the native mouse hearts. The mouse hearts are at least twice as stiff as the microtissues. Stiffness of the microtissues could be increased by increasing the cross-linking density with transglutaminase [41], a process that also happens in vivo with aging of the heart [42] or by changing composition of the hydrogel by adding other matrix components.

### Fibroblast Density Rather than Collagen Content Weakens Cardiomyocyte Contractile Function

In cardiac fibrosis, synchronized beating of the heart is often hampered. However, it remains unclear how fibroblast proliferation and accumulation of matrix influence cardiac contractility on the cellular level. Next to the synthesis of ECM, cardiac fibroblasts play an important role in the maintaining of normal cardiac functions. The fibroblasts also play an important role in cell-cell communication not only with cardiomyocytes but also with endothelial cells and other fibroblasts. These contacts play an important role in electrophysiological properties, in secretion of cytokines and growth factors, and in matrix regeneration and degradation under influence of the production metalloproteinases and their inhibitors [43]. Therefore, cardiomyocyte functionality was assessed by measuring dynamic contraction force and beating frequency of the microtissues representing the different aspects of fibrosis. Our results demonstrated a significant decrease in beating frequency when the number of fibroblasts increased. This correlates with previous 2D research, which demonstrated a decreased conduction velocity with increased fibroblast proliferation [44]. Furthermore, studies by others have shown that fibroblasts can electrically connect to cardiomyocytes via gap junctions [45, 46]. Although the gap junction protein connexin43 was present in all cardiac microtissues, the 3D distribution was random and not organized as in intercalated disks (data not shown). Therefore, our results could not be directly linked to gap junction formation between cardiomyocytes and fibroblasts. Nevertheless, in 2D, it was shown that cardiomyocytes beat much faster when cultured on fibroblasts devoid of connexin43 than on wild-type fibroblasts that do express these gap junction proteins [47]. Although it has frequently been reported that fibroblast proliferation inhibits the contractility of cardiomyocytes, here, we demonstrated a threshold value of 50% fibroblasts above which synchronized beating was severely hampered.

Mimicking cardiac fibrosis by increasing collagen content of the microtissues, surprisingly, showed no effect on contraction force or beating frequency. This implies that the cardiomyocytes were still able to exert the same force of contraction, even though there was more collagen in between neighboring cells. These results might be explained by the fact that only collagen content but probably not the cross-linking density was increased and therefore did not affect tissue stiffness [39].

By separately increasing collagen content and fibroblast density in our in vitro model of cardiac fibrosis, we were able to show that fibroblast density has a more destructive influence on cardiomyocyte contraction than collagen content.

### Limitations and Future Perspectives

The microtissue platform described in this study is designed to expand our knowledge about clinically relevant problems regarding myocardial fibrosis and improve currently available therapies. However, translating observations from a micrometer scale cardiac tissue towards a four-chambered heart or even the other way around has its limitations. Differences in measurement techniques complicate correlating the outcomes of in vitro and in vivo studies. Although, in this study, microtissues were created with a ratio of cardiomyocytes to fibroblasts which closely mimics the native tissue composition, changes in cell density or total number of cells present in the gel will influence cell behavior, matrix modulation, and therefore the overall outcome. This study is a first step, and further research is need to keep improving the microtissue model for cardiac fibrosis to fully develop this high potential system into a controllable and reliable screening tool.

## Conclusion

An in vitro tissue model is always a simplified reproduction that can never reach the complexity found in native tissue. It is exactly this simplicity that allows us to separately study and understand the different aspects of a complex condition such as cardiac fibrosis. In this study, we mimicked several aspects of cardiac fibrosis by increasing either the collagen content or the fibroblast density in our tissue model of cardiac fibrosis. In this way, we were able to study the effect of these aspects on cardiomyocyte functionality individually, which is not possible in vivo, and showed that increased fibroblast density has a more detrimental effect on cardiomyocyte functionality than increased collagen content. Because our tissue model can be easily adapted to mimic other aspects of cardiac fibrosis, we anticipate that it can be more widely used to gain further insight into the mechanisms behind cardiac fibrosis and contribute to development and testing of new anti-fibrotic therapies and thereby reduce in vivo testing.

## Electronic Supplementary Material

ESM 1 (DOC 48 kb)

## Abbreviations

ECM: Extracellular matrix
TAC: Transverse aortic constriction
HYP: Hydroxyproline
μTUG: Microfabricated tissue gauge
FB: Fibroblast
