# Supplementary material for: Mimicking Cardiac Fibrosis in a Dish: Fibroblast Density Rather than Collagen Density Weakens Cardiomyocyte Function
Source: J Cardiovasc Transl Res. 2017 Mar 9;10(2):116–27. doi: 10.1007/s12265-017-9737-1 (PMC5437129; doi:10.1007/s12265-017-9737-1)
Supplement: Supplementary file 1 — (DOC 48 kb) [file 12265_2017_9737_MOESM1_ESM.doc]

## Supplementary material and methods

## Animal models and tissue preparation

C57Bl/10ScSnJ (control) and *mdx* (C57Bl/10ScSn-DMD^mdx^/J) male mice at 10 months were sacrificed by cervical dislocation and hearts were isolated [3, 4]. To induce pressure overload (TAC) in the left ventricle, 11 weeks old male C57BL/6 were anesthetized with 2.5%vol/isoflurane in 0.2 L/min O_2_ and 0.2 L/min medical air and intubated for mechanical ventilation. To maintain the body temperature at 37^o^C, animals were placed on a heating pad. Buprenorphine (0.1mg/kg subcutaneously injected) was administered for analgesia. Surgical procedures were performed as described previously. Briefly, a small incision was made above the first intercostal space and just lateral form the sternum. The aortic arch was tied off (6-0 silk suture) together with a 27G (Ø0.42 mm) needle between the brachiocephalic artery and the left common carotid artery. Blood flow was restored by immediate removal of the needle [5, 6]. Nine weeks after surgery hearts were excised as well as the hearts from age matched male controls (21 weeks). Hearts were perfused with PBS and cryopreserved in medium (DMEM High glucose, Gibco) supplemented with 10% fetal bovine serum and 10% dimethylsulfoxide. The freezing process minimized the formation of ice crystals within the tissue by freezing at a rate of -1^o^C/minute. Frozen hearts were stored at -80^o^C before further examination. Before analysis, cyropreserved hearts were quickly thawed and washed with cold PBS.

## Neonatal cardiac cell isolation and culture

Mouse neonatal cardiomyocytes and cardiac fibroblasts were isolated from 1–3 day old C57/BL6 mouse hearts as described previously [1, 2]. Briefly, the hearts were incubated overnight in Trypsin (Sigma) before using three-minute collagenase A (Sigma) incubation steps to dissociate the tissue. Enriched cardiomyocytes were cultured in six-well plates coated with 1% gelation and 10µg/ml fibronectin (Tebu-Bio) at a density of 5x10^4^ cells/cm^2^. Cardiac fibroblasts were cultured until confluent before they were split 1:3. When cultured until confluent again, the cells were frozen and stored for next experiments. Freezing medium for cardiac fibroblasts consisted of culture medium with an additional 45% heat-inactivated fetal bovine serum and 10% DMSO. After thawing, fibroblasts were seeded in culture flasks coated with 1% gelatin and 10µg/ml fibronectin.

**Stiffness measurement**

To determine local material stiffness of cardiac tissue samples, micro-indentation was performed. Cryopreserved hearts (n=2-4 per group) were quickly thawed and cut parallel to the myocardial wall with a cryotome in 500µm thick slices. Due to considerable variation per location, three different locations per heart sample were investigated and two consecutive indentation tests were performed per location [7, 8]. A spherical indenter with a diameter of 2mm was used to indent the tissue slices. The experimental force-depth curves were fitted to numerical simulations of indentation to determine the Young’s Modulus using Sepran and Matlab as described before [7, 8]. Isotropic neo-Hookean behavior and homogeneity of the heart samples was assumed.

Stiffness of the different hydrogel compositions that were used for microtissue engineering was determined using a Piuma nanoindenter (Optics11, The Netherlands). Tissues consisted of 100µl of hydrogel with 1x10^6^ cells/ml. For microtissues with extra cardiac fibroblasts the same cell densities as described above were used. For microtissues with increased collagen content, final concentrations as described above were used. After 30 minutes of gelation, medium was added to the constructs. Nanoindentation was applied at day two of culture using a glass spherical indenter with a radius of 44µm and a spring constant k=0.52N/m. Samples were indented for 10µm on three different spots per sample. The Young’s modulus (E-modulus) was calculated from the force-displacement curves by the Piuma software based on the method by Oliver and Pharr, thereby using the part of the unloading curve between 80-90% [9].

**Histology, immunofluorescence and image analysis**

For histology, hearts were fixed in 3.7% formaldehyde in PBS for 24 hours at 4^o^C and further processed for paraffin immunohistological investigation. Subsequently, 5 µm serial sections were mounted on polylysine coated microscopy slides. To confirm presence of collagen, serial sections were stained with picrosirius red according to standard histological procedures and analyzed using a Zeiss light microscope. Semi-quantification of picrosirius red staining was performed using ImageJ by comparing the percentage of fibrosis to the total amount of tissue within images. A minimum of 6 images per ventricle throughout the heart were analyzed.

Microtissues were fixed in 3.7% formaldehyde for 10 minutes at day 7 of culture and stored in PBS before immunofluorescence staining.

For immunofluorescence, sections and microtissues were stained according to standard immunohistochemistry protocols (Supplementary table 1). Sections were mounted with mowiol and analyzed using a Zeiss fluorescence microscope. Microtissues were analyzed using confocal microscopy performed on a Zeiss 2-photon confocal laser scanning microscope. To determine the percentage of cardiac fibroblasts in the microtissues, α-actinin and vimentin positive cells were counted in ImageJ using two images per sample with a 40x magnification.

**Biochemical assay**

Heart samples were analyzed with a biochemical assay for the amount of hydroxyproline (HYP), the major component of collagen [10]. Left and right heart sides were divided in three parts and samples were lyophilized and digested overnight in papain solution (100 mM phosphate buffer [pH=6.5], 5 mM L-cystein, 5 mM EDTA, and 140 µg papain per mL (all Sigma)) at 60^o^C. Digestion supernatant was collected, centrifuged for 10 min at 12000 rpm and stored at -20^o^C for further analysis. Undigested heart tissue was lyophilized and subjected to a second digestion overnight. Samples were centrifuged and analyzed at the same time as the first digestion samples.

**Tissue compaction and force measurement**

After seeding, cells compacted the matrix around the microposts. Beating of the cells caused displacement of the microposts. Movies were recorded at day two using a high speed camera mounted on a Zeiss observer microscope with a 10x magnification. Tissue width was measured for each sample using the first frame of the recorded movie. Compaction was calculated as the percentage of decrease in tissue width in the middle of the tissue. The distance between the micoposts (325µm) was used a reference for 0% compaction.

Dynamic contraction force and beating frequency of the microtissues was calculated from the micropost displacements as described previously [1]. Displacement of the microposts was tracked using feature detection software in Mathemica and Matlab. The displacement-force relationship of the microposts with a spring constant of 0.7µN/µm [1] was then used to calculate the dynamic contraction forces of the microtissues. Beating frequency was deducted from the Fourier Spectrum of the signal.

**Statistical analysis**

All results were expressed as mean ± standard deviation. Statistical analysis was performed with GraphPad Prism software. A non-parametric Mann-Whitney test or Kruskal-Wallis test with Dunns post-hoc test was performed for data with no Gaussian distribution. A one-way ANOVA with Bonferroni correction for multiple comparisons was applied to data with a normal Gaussian distribution. Statistical significance was defined when P <0.05.

## Supplementary table

**Table 1.** Antibodies used for immunohistochemistry of native and engineered cardiac tissue

| **Antigen** | **Source** | **Isotype** | **Species** | **Antigen Retrieval** | **Blocking agent** | **Dilution solution** | **Dilution** | **Label** |
| --- | --- | --- | --- | --- | --- | --- | --- | --- |
| ***Collagen I*** | Abcam (AB34710) | IgG | Rabbit | Citrate (pH6.0) | 5% protifar | 0.5% protifar | 1:100 | - |
| ***Collagen III*** | Abcam (AB7778) | IgG | Rabbit | Pepsin (pH2.0) | 5% protifar | 0.5% protifar | 1:100 | - |
| ***Fibronectin*** | SA (F3648) | IgG | Rabbit | Tris-EDTA (pH9.0) | 10% HS | 1% HS | 1:200 | - |
| ***α-Actinin*** | SA (A7811) | IgG1 | Mouse | - | 10% HS | 1% HS | 1:800 | - |
| ***Vimentin*** | CS  (5741) | igG | Rabbit | - | 10% HS | 1% HS | 1:400 |  |
| ***Phalloidin*** | SA (49409) | - | - | - | 10% HS | 1% HS | 1:200 | Atto 488 |
| ***Rabbit IgG*** | IG (A31572) | IgG | Donkey | - | - | - | 1:300 | Alexa Fluor 555 |
| ***Mouse IgG1*** | MP (A21127) | IgG1 | Goat | - | - | - | 1:300 | Alexa Fluor 488 |

Abbreviations used in this table: SA, Sigma Aldrich; IG, Invitrogen; MP, Molecular Probes; CS, Cell Signalling; HS, horse serum.

## Supplementary references

[1] van Spreeuwel ACC, Bax NAM, et al (2014) The influence of matrix (an)isotropy on cardiomyocyte contraction in engineered cardiac microtissues. Integr Biol (Camb) 6(4):422-9 doi: 10.1039/c3ib40219c

[2] Weeke-Klimp A, Bax NAM, et al (2010) Epicardium-derived cells enhance proliferation, cellular maturation and alignment of cardiomyocytes. J Mol Cell Cardiol 49(4):606-16 doi: 10.1016/j.yjmcc.2010.07.007

[3] Verhaart IE, van Duijn RJ, et al (2012) Assessment of cardiac function in three mouse dystrophinopathies by magnetic resonance imaging. Neuromuscul Disord 22(5):418-26 doi: 10.1016/j.nmd.2011.10.025

[4] van Putten M, van der Pijl EM, et al (2014) Low dystrophin levels in heart can delay heart failure in mdx mice. J Mol Cell Cardiol 69:17-23 doi: 10.1016/j.yjmcc.2014.01.009

[5] van Deel ED, de Boer M, et al (2011) Exercise training does not improve cardiac function in compensated or decompensated left ventricular hypertrophy induced by aortic stenosis. J Moll Cell Cardiol 50(6):1017-25 doi: 10.1016/j.yjmcc.2011.01.016

[6] van Nierop BJ, Coolen BF, et al (2014) Myocardial perfusion MRI shows impaired perfusion of the mouse hypertrophic left ventricle. Int J Cardiovasc Imaging 30(3):619-28 doi: 10.1007/s10554-014-0369-0

[7] Cox MA, Driessen NJ, Bouten, CV, Baaijens FP (2006) Mechanical characterization of anisotropic planar biological soft tissues using large indentation: a computational feasibility study. J Biomech Eng 128(3):428-36

[8] Chai CK, Akyildiz AC, et al (2013) Local axial compressive mechanical properties of human carotid atherosclerotic plaques-characterization by indentation test and inverse finite element analysis. J Biomech 46(10):1759-66 doi: 10.1016/j.biomech.2013.03.017

[9] Oliver WC, Pharr GM (2004) Measurement of hardness and elastic modulus by instrumented indentation: Advances in understanding and refinements to methodology. J Mater Res 19(1):3-20

[10] Huszar G, Maiocco J, Naftolin F (1980) Monitoring of collagen and collagen fragments in chromatography of protein mixtures. Anal Biochem 105(2):424-9
